# Wnt/β-Catenin Inhibition by CWP232291 as a Novel Therapeutic Strategy in Ovarian Cancer

**DOI:** 10.3389/fonc.2022.852260

**Published:** 2022-05-12

**Authors:** Wenyu Wang, Untack Cho, Anna Yoo, Chae-Lim Jung, Boyun Kim, Heeyeon Kim, Juwon Lee, HyunA Jo, Youngjin Han, Myoung-Hyun Song, Ja-Oh Lee, Se Ik Kim, Maria Lee, Ja-Lok Ku, Cheol Lee, Yong Sang Song

**Affiliations:** ^1^Interdisciplinary Program in Cancer Biology, Seoul National University College of Medicine, Seoul, South Korea; ^2^Cancer Research Institute, Seoul National University College of Medicine, Seoul, South Korea; ^3^Drug Discovery Center, JW Pharmaceutical Corporation, Seoul, South Korea; ^4^Department of Biosafety, College of Life and Health Science, Kyungsung University, Busan, South Korea; ^5^WCU Biomodulation, Department of Agricultural Biotechnology, Seoul National University, Seoul, South Korea; ^6^Department of Biomedical Sciences, Seoul National University College of Medicine, Seoul, South Korea; ^7^Korean Cell Line Bank, Laboratory of Cell Biology, Cancer Research Institute, Seoul National University College of Medicine, Seoul, South Korea; ^8^Department of Obstetrics and Gynecology, Seoul National University College of Medicine, Seoul, South Korea; ^9^Department of Pathology, Seoul National University College of Medicine, Seoul, South Korea

**Keywords:** Wnt/β-catenin, CWP232291, targeted therapy, organoids, ovarian cancer

## Abstract

The poor prognosis of ovarian cancer patients mainly results from a lack of early diagnosis approaches and a high rate of relapse. Only a very modest improvement has been made in ovarian cancer patient survival with traditional treatments. More targeted therapies precisely matching each patient are strongly needed. The aberrant activation of Wnt/β-catenin signaling pathway plays a fundamental role in cancer development and progression in various types of cancer including ovarian cancer. Recent insight into this pathway has revealed the potential of targeting Wnt/β-catenin in ovarian cancer treatment. This study aims to investigate the effect of CWP232291, a small molecular Wnt/β-catenin inhibitor on ovarian cancer progression. Various *in vitro*, *in vivo* and *ex vivo* models are established for CWP232291 testing. Results show that CWP232291 could significantly attenuate ovarian cancer growth through inhibition of β-catenin. Noticeably, CWP232291 could also s suppress the growth of cisplatin-resistant cell lines and ovarian cancer patient-derived organoids. Overall, this study has firstly demonstrated the anti-tumor effect of CWP232291 in ovarian cancer and proposed Wnt/β-catenin pathway inhibition as a novel therapeutic strategy against ovarian cancer.

## Introduction

Ovarian cancer is the most lethal gynecologic malignancy globally, ranking the eighth leading cause of cancer deaths among women (1). Ovarian cancer can be mainly grouped into epithelial, stromal and germ cell cancer according to the cell origin, among which the epithelial ovarian cancer accounts for over 90% of all types (2). Epithelial ovarian cancer covers a wide range of histological features and is further categorized into four subtypes: serous, endometrioid, clear cell and mucinous subtypes. Most epithelial ovarian cancer patients are primarily diagnosed at advanced stages because of the lack of characteristic symptoms and early diagnostic biomarkers (2, 3). Currently, the standard treatment strategy for ovarian cancer is debulking surgery followed by platinum and taxol-based chemotherapy. However, more than 70% of the patients develop relapse due to chemoresistance within three years (4, 5). Recently, the implementation and optimization of maintenance strategies fundamentally has influenced ovarian cancer treatment. More targeted therapies including vascular endothelial growth factor A (VEGF‐A) inhibitors and poly ADP-ribose polymerase (PARP) inhibitors have become new therapeutic options in managing ovarian cancer (6, 7).

Wnt signaling pathway is implicated in a wide range of biological processes. This pathway is classically subdivided into the canonical pathway, namely Wnt/β-catenin signaling and non-canonical pathway including Wnt/planar cell polarity (PCP) pathway and Wnt/Ca2^+^ pathway (8, 9). Wnt/β-catenin pathway orchestrates various aspects of cancer development and progression. The aberrant regulation of Wnt/β-catenin pathway is significantly involved in cancer proliferation, metastasis, stemness, chemoresistance and immune responses, etc. (10–13). In the absence of Wnt ligands, the expression of β-catenin remains at a low level in that β-catenin can be constantly phosphorylated with the assembly of the destruction complex, which subsequently leads to the ubiquitination and proteasomale degradation of β-catenin (14). However, β-catenin signaling can be activated in the presence of Wnt ligands. To be specific, Wnt ligands bind to the frizzled (FZD) family, which induces the accumulation of β-catenin in the cytoplasm by disrupting the destruction complex (15). Subsequently, β-catenin is translocated into the nucleus to activate the T cell factor/lymphoid enhancer factor-1 (TCF/Lef1) transcription complex (16, 17). Wnt/β-catenin pathway has been reported to play a vital role in various solid tumors and hematological malignancies (15, 18, 19). Growing evidence has illustrated that Wnt/β-catenin pathway is hyperactivated in ovarian cancer, exerting essential functions in ovarian cancer stemness, drug resistance, metastasis, etc. For example, β-catenin was shown to play an important role in maintaining ovarian cancer stem cell phenotype through regulation of ALDH1A1 (20). S. To et al. also reported that β-catenin as a critical factor in promoting ovarian cancer metastasis by affecting miRNA biogenesis (21). In addition, forced activation of canonical Wnt signaling *via* WNT3A promoted HGSOC, poly (ADP)-ribose polymerase inhibitors (PARPi) resistance in ovarian cancer (22). Targeting Wnt/β-catenin pathway has risen to be a novel therapeutic strategy in cancer treatment.

Several antibodies or small molecules targeting Wnt/β-catenin pathway are currently in clinical trials (23–25). CWP232291 is a small molecular prodrug that can be converted into its active form CWP232204. CWP232204 can induce endoplasmic reticulum (ER) stress, leading to the activation of caspases, a decrease of β-catenin and inhibition of β-catenin-mediated transcriptional activity. In addition, CWP232204 can promote the apoptosis of cancer cells through binding to Src-Associated substrate in Mitosis of 68 kDa (Sam68). This function can also be partially attributed to the suppression of some anti-apoptotic genes like survivin. CWP232291 has been reported to suppress tumor growth in various cancers (26–28). There is a phase 1 clinical trial of CWP232291 ongoing in patients with myeloid leukemia and myelodysplastic syndrome (28). However, the function of CWP232291 in ovarian cancer is still under elucidated. In this study, we investigated the effect of CWP232291 in ovarian cancer using various experimental models and shed new light on the novel therapeutic value of Wnt/β-catenin pathway inhibition.

## Methods

### Clinical Sample Collection

This study was approved by the Institutional Review Board of Seoul National University Hospital (IRB No. 1508-165-699). Ovarian cancer tissue samples were collected with the consent of patients. All the experiments were performed in compliance with the Declaration of Helsinki. Twenty tissue samples were used for organoid experiments and fifty-six tissue samples were used for immunohistochemistry. Patient clinical information for organoid establishment and immunohistochemistry was relatively summarized in **Supplementary Table S1**.

### Bioinformatics Analysis

Gene Set Enrichment Analysis (GSEA) was performed using the GSEA tool provided by the Broad Institute (http://software.broadinstitute.org/gsea). Datasets for (GSEA) were downloaded from GEO database (https://www.ncbi.nlm.nih.gov/gds). Kaplan–Meier survival data were generated with multiple datasets using the publicly accessible online tool KM-plotter (http://kmplot.com/analysis/). Mutational signatures of ovarian cancer patients were obtained from the COSMIC: Catalogue of Somatic Mutations in Cancer (http://cancer.sanger.ac.uk/cosmic).

### Cell Culture

Human ovarian cancer cell lines used in this study were obtained from the American Type Culture Collection (Rockville, MD, USA) and Korean Cell Line Bank (Seoul, Korea). Ovarian cancer cells were maintained in RPMI1640 (WelGENE, Seoul, Korea) supplemented with 10% fetal bovine serum (FBS; Gibco, MD, USA) and 100 μg/mL penicillin-streptomycin (Invitrogen, CA, USA) in 37°C, 5% CO2.

### Cell Viability Assay

MTT Assay was carried out to detect the viability of ovarian cancer cells. Cells were seeded in 96-well plates while organoids embedded in Matrigel were seeded in 24-well plates. For cell viability assessment, 50 μl of MTT reagent was applied to each well without discarding the culture media. After 3h of incubation, 100 μl DMSO was added after discarding the culture media to dissolve formazan crystals. For organoid viability analysis, 500 μl of MTT reagent was applied to each well first. After 3h of incubation, 200 μl of DMSO was added and the Matrigel was dissociated by pipetting. The plates were placed on an orbital shaker and incubated for 30 min at room temperature. The optical density of each well was detected at 540 nm with a Multi-Scan Spectrum (Thermo Scientific, NH, USA).

### Western Blotting

Western blotting assay was carried as previously described (29). Briefly, ovarian cancer cells were harvested and lysed in the lysis buffer comprised of 2X lysis buffer (10 mM Tris-HCl, 150 mM NaCl, 1 mM EGTA and 1 mM EDTA), 1% Triton X-100, 1 mM phenylmethylsulfonyl fluoride, 1 mg sodium deoxycholate, EDTA-free protease inhibitor cocktail, and 1 mM Na3VO4 for 30 min. Then the buffer was centrifuged at 13,000 rpm, 4°C for 20 min and the supernatant was collected for concentration determination by a BCA Protein Assay kit (Thermo Scientific, MA, USA). Subsequently, 10μg of each protein sample was loaded and separated in the SDS-PAGE. Gels were transferred to nitrocellulose membranes. After blocking in 5% skim milk, the membranes were subsequently incubated with primary antibodies and horseradish peroxidase-conjugated secondary antibodies (1:5000). Lastly, signals were detected using a Westsave Western Blotting Detection Kit (AbFrontier, Seoul, South Korea). Primary antibodies used in this study were β-catenin (1:1000; Cell Signaling: #9562), active β -catenin (1:1000; Cell Signaling #8814), cleaved caspase3 (1:1000; Cell Signaling #9661), PARP-1:1000; Cell Signaling #9542) and GAPDH (1:2500; Santa Cruz: sc-365062).

### Immunohistochemistry (IHC)

IHC assay was performed using the 4μm thick tissue microarray (TMA) sections with a Benchmark autostainer (Ventana, Tucson, AZ, USA) using a Ventana Ultra-View Universal DAB Detection Kit (Ventana, Tucson, AZ, USA) according to the manufacturer’s instructions. Briefly, deparaffination and rehydration of the sections was performed firstly and antigen retrieval was then done at 100°C for 24 min. Slides were treated with inhibitors to endogenous peroxidase followed to incubation with B catenin primary antibody (Abcam: ab32572; diluted at 1:100) in 16 min at 37°C. The visualization was done using the HRP secondary antibody and the DAB detection kit. The slides were counterstained with hematoxylin for 8 min at 37°C. Slides were scanned for analysis using the Aperio AT2 Scanner (Aperio Technologies, Vista, USA) and digitalized using Aperio ImageScope software.

### Organoid Establishment

Organoids were established with ovarian cancer tissues as previously reported (30). Briefly, ovarian cancer tissues were minced using a razor blade mechanistically and incubated in the diluted dispase (Gibco, MD, USA) in 37°C for 1h. Then, the undigested tissue debris was eliminated by filtering with a 100μm cell strainer. Then the cells isolated were embedded in the in phenol red-free Matrigel Growth Factor Reduced Basement Membrane Matrix (BD Bioscience, CA, USA) and cultured in Advanced DMEM/F12 (Gibco, MD, USA) supplemented with HEPES (10mM; Gibco, Gaithersburg, MD, USA), 1× GlutaMax (Gibco, Gaithersburg, MD, USA), 1× N2 (Invitrogen, CA, USA), 1× B27 (Invitrogen, CA, USA), β-Estradiol (1 mM; Sigma-Aldrich, St. Louis, USA), nicotinamide (1 mM; Sigma-Aldrich, St. Louis, USA), recombinant human Noggin (10 ng/mL; PeproTech, Rocky Hill, NJ, USA), recombinant R-Spondin1 (10 ng/mL; PeproTech, Rocky Hill, NJ, USA), EGF (10 ng/mL; Invitrogen, CA, USA), FGF2 (10 ng/mL; PeproTech, Rocky Hill, NJ, USA), FGF10 (10 ng/mL; PeproTech, Rocky Hill, NJ, USA), Y-27632 dihydrochloride (10 μM; Sigma-Aldrich, St. Louis, USA), SB431542 (0.5 μM; Sigma-Aldrich, St. Louis, USA), and N-acetylcysteine (1mM; Sigma-Aldrich, St. Louis, USA).

### Organoid Staining

Organoids were harvested and washed twice with PBS. Fixation/Permeabilization solution kit (BD Biosciences, CA, USA) was added to the organoids and incubated for 4 °C for 20 min. Then the kit washing buffer was added to resuspend organoids and let them settle at the bottom of the tube by gravity. The supernatant was removed and kit washing buffer was added to repeat this process. Subsequently, organoids were incubated in 1% BSA diluted in PBS-T (1.25% Triton X 100 in PBS) at room temperature for 45 min and washed with PBS-T for three times. Recombinant Alexa Fluor^®^ 488 Anti-Ki67 antibody (Abcam, Cambridge, UK), Hoechst33342 (Invitrogen, CA, USA) and F-actin diluted in phalloidin (Sigma Aldrich, USA) was used for immunostaining. Imaging was performed using the confocal microscopy LSM800 (EVOS, USA).

### Animal Experiments

All the protocols of animal experiments were approved by the Seoul National University Institutional Animal Care and Use Committee (SNU-151002-1). The mice were housed in a specific pathogen-free facility with 12h light/dark cycles and ad libitum access to food and water. All the experiments were carried out in accordance with the guidelines. The human ovarian cancer cell line PA-1 was injected subcutaneously into the right dorsal flanks of the female BALB/C nude mice (OrientBio, Seoul, Korea) to develop tumor xenograft. Thirteen days after inoculation, mice were randomly divided into the experimental and control group with five mice included in each group. The experimental group received the intravenous (tail vein) injection of CWP232291 (100mg/kg) in distilled water while the control group received an injection of the vehicle only for ten days with an interval of two days. The tumor size was measured with a caliper every other day and calculated using the formula: 0.5 × length × (width)^2^.

### Data Analysis

Data were presented as mean ± SEM using the GraphPad Prism software. The statistical analysis was done using Student’s t-test and one-way ANOVA analysis with a p value <0.05 considered statistically significant.

## Results

### Wnt/β-Catenin Signaling Dysregulation Is Closely Associated With Ovarian Cancer Progression and Patient Prognosis

**Figure 1:**
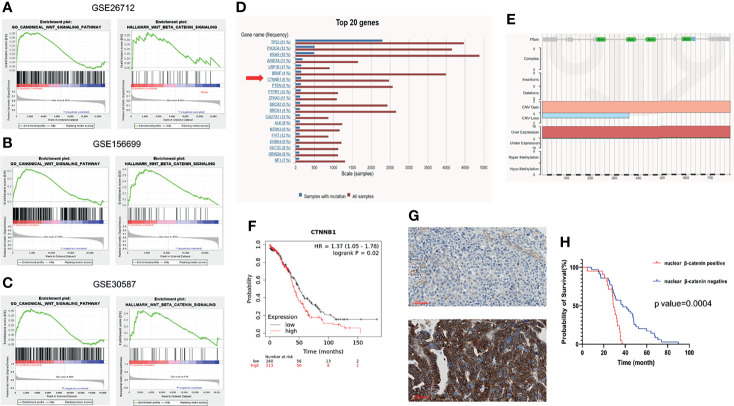
Wnt/β-catenin pathway in ovarian cancer progression. GSEA was performed using the GSEA tool provided by the Broad Institute (http://software.broadinstitute.org/gsea). Enriched plots were generated to compare the expression of a set of genes in GO canonical Wnt signaling and Hallmark Wnt/β-catenin signaling between different groups of datasets (X axis means the gene hits and Y axis means the enrichment score. Red part means genes that positively correlated with the pathway and blue part means genes that negatively correlated with the pathway). **(A)** The gene expression profiling of 185 primary ovarian cancer tissues was included in GSE26712 dataset. Samples were divided according to the overall survival (OS) of patients into the long (OS >= 36 months) and short survival group (OS < 36 months). Wnt/β-catenin pathway was shown to be enriched in the short OS group than the long OS group by GO analysis (left) and Hallmark analysis (right). **(B)** The gene expression profiling of 88 high-grade serous ovarian cancer tissues was included in GSE156699 dataset. Samples were divided into the chemotherapy responder group and non-responder group according to patients’ response to chemotherapy. Wnt/β-catenin pathway was enriched in the chemotherapy non-responder group by GO analysis (left) and Hallmark analysis (right). **(C)** The gene expression profiling of 18 paired ovarian cancer primary tissues and omental metastases was included in GSE30587 dataset. Wnt/β-catenin pathway was enriched in omental metastases than primary sites by GO analysis (left) and Hallmark analysis (right). **(D)** Top 20 frequently mutated genes were listed in ovarian cancer in COSMIC (https://cancer.sanger.ac.uk/cosmic/). Among these, CTNNB1 mutation frequency accounted for 6% among all ovarian cancer patients. **(E)** Most of the CTNNB1 mutation types were copy number variation (CNV) gain or gene overexpression. CTNNB1 expression was negatively related to the overall survival of ovarian cancer patients by Kaplan-Meier Plotter (https://kmplot.com/analysis/). Nuclear β-catenin expression was significantly associated with the survival of ovarian cancer patients **(F)**. Nuclear β-catenin expression was assessed using the high-grade serous ovarian cancer tissue microarray (N=56). Samples were scored as negative (above) or positive (below) according to the β-catenin staining intensity in the nucleus **(G)**. **(H)** The positive nuclear β-catenin expression was correlated with a worse overall survival (Log-rank test, p value=0.0004).

To explore the functional role of Wnt/β-catenin signaling in ovarian cancer, GSEA was performed using GEO public datasets. The expression of a set of genes in GO canonical Wnt signaling and Hallmark Wnt/β-catenin signaling between two groups was compared and the enriched plots were generated by GSEA. The gene expression profiling of 185 primary ovarian cancer tissues was included in the GSE26712 dataset. Samples were divided according to the overall survival (OS) of patients into the long survival (OS >= 36 months) and short survival group (OS < 36 months). The analysis illustrated that Wnt/β-catenin signaling was significantly enriched in the short survival group(**Figure 1A**). Similarly, samples included in the GSE156699 dataset were divided into the chemotherapy responder group and the non-responder group. The enrichment Wnt/β-catenin pathway was detected in the non-responder group (**Figure 1B**). In addition, eighteen samples from nine matched pairs of primary ovarian tumors and metastases from the omentum were included in GSE30587 dataset. Wnt/β-catenin signaling enrichment was also observed in metastatic sites than primary sites (**Figure 1C**).

The most frequent genetic alteration in Wnt/β-catenin signaling in ovarian cancer is in the CTNNB1, the gene of β-catenin (31). CTNNB1 mutation often leads to β-catenin accumulation in the nucleus, subsequently promoting the transcriptional activity of its target genes. Herein, we investigated CTNNB1 mutation in 5942 patients with different subtypes of ovarian cancer in COSMIC, a somatic mutation database (http://cancer.sanger.ac.uk/cosmic). The top twenty most common mutated genes were presented in **Figure 1D**. The results showed that CTNNB1 mutation existed in around 6% of ovarian cancer patients; among that the most common CTNNB1 gene alterations were copy number variation (CNV) gain and gene overexpression (**Figure 1E**). Both of these two alterations commonly lead to the gain-of-function of β-catenin. Furthermore, the CTNNB1 expression at the transcriptional level was negatively associated with the survival of ovarian cancer patients (**Figure 1F**). The bioinformatic analysis depicted that the aberrant activation of Wnt/β-catenin plays a fundamental role in ovarian cancer progression, implying the potential therapeutic significance of Wnt/β-catenin pathway inhibition in ovarian cancer management. Wnt/β-catenin signaling is turned on when β-catenin is translocated into the nucleus, facilitating the transcriptional activity of target genes. The expression of β-catenin in different subcellular locations is critical in tumor progression. In ovarian cancer, previous studies have shown that the nuclear and membranous expression of β-catenin was both related to patient survival and could also be a predictive marker of chemotherapy response (32, 33) In this study, the ovarian cancer tissue microarray (TMA) of primary high-grade serous ovarian cancer tissues from 56 patients was used to detect the relationship between nuclear β-catenin expression and patient survival (**Supplementary Table S1**). The β-catenin nuclear expression was scored as negative or positive according to the staining intensity (**Figure 1G**). The survival analysis elucidated that patients with positive nuclear β-catenin expression had a remarkably shorter OS than those with negative nuclear β-catenin expression (**Figure 1H**).

### CWP232291 Suppresses Ovarian Cancer Growth *In Vitro* and *In Vivo*

**Figure 2:**
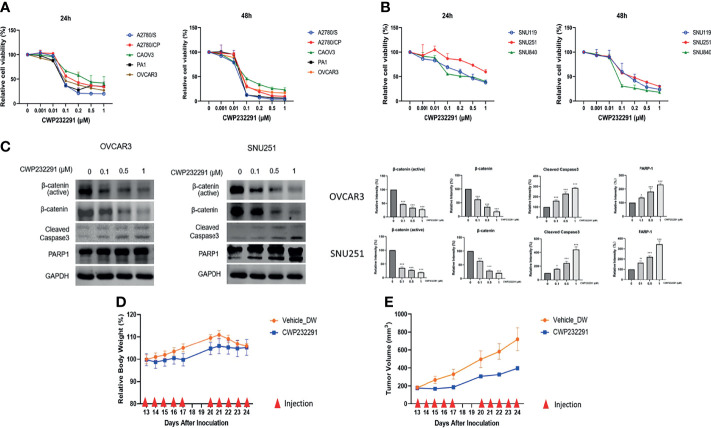
CWP232291 suppressed the growth of various ovarian cancer cell lines through β-catenin inhibition. Five ATCC cell lines, A2780/S, A2780/CP, CAOV3, PA1 and OVCAR3 **(A)** and three SNU cell lines, SNU119, SNU251, SNU840 **(B)** were used in this study. Cells were treated with different doses (0, 0.001, 0.01, 0.1, 0.2, 0.5, 1.0 µM) of CWP232291 for 24h and 48h. Cell viability was measured through MTT assay. **(C)** The representative ATCC cell line OVCAR3 and SNU cell line SNU251 were treated with different doses of CWP232291 (0, 0.1, 0.5, 1µM) for 24h and the expression of active β-catenin form, total β-catenin form and apoptotic markers was determined by Western blotting. CWP232291 inhibited tumor growth in the *in vivo* mouse model. **(D)** The tumor size was measured and calculated using the formula: 0.5 × length × (width)^2^. **(E)** The body weight of mice was also examined together with the tumor size. Results were shown as mean ± SEM (*p ≤ 0.05, **p ≤ 0.01, ***p ≤ 0.001).

The aberrant activation of Wnt/β-catenin signaling in ovarian cancer drove us to investigate the effect of Wnt/β-catenin inhibition in cancer progression. CWP232291, a small molecule β-catenin inhibitor was used in this study. In total, eight ovarian cancer cell lines of different histologic subtypes (**Supplementary Table S2**) were applied to examine the effect of CWP232291 in ovarian cancer. Cancer cells were treated with CWP232291 in different doses for 24h and 48h, respectively. The cell viability assay result showed that CWP232291 treatment significantly hindered the growth of cancer cells (**Figures 2A, B**). The IC50 of CWP232291 of cell lines was summarized in **Supplementary Table S3**. Meanwhile, CWP232291 treatment could reduce the expression of the total form and active form of β-catenin (a non-phosphorylated form of β-Catenin when residues Ser33, Ser37, and Thr41 are not phosphorylated) in a dose-dependent manner (**Figure 2C**). In addition, the expression of apoptotic markers including cleaved caspase3 and PARP1 was also significantly increased by CWP232291 treatment (**Figure 2C**). The above data demonstrated that CWP232291 could hamper ovarian cancer cell growth by inhibiting β-catenin and inducing cell apoptosis.

Tumor xenograft models can mimic the tumor growth condition *in vivo* serve as an exemplary model in the pre-clinical research. To further delve into the anti-tumor effect of CWP232291, we established the tumor xenograft mouse model with the subcutaneous injection of cancer cell line PA-1. PA-1 was chosen for xenograft development in that it showed a good response to CWP232291 and has a strong proliferative ability. PA-1 cells were injected subcutaneously into the right dorsal flanks of the female BALB/C nude mice to develop tumor xenograft. Thirteen days after inoculation, mice were randomly divided into the experimental and control group with five mice in each group. The experimental group received the intravenous injection of CWP232291 (100mg/kg) in distilled water while the control group received the injection of the vehicle only for ten days with an interval of two days. The result demonstrated that tumor xenograft growth was significantly inhibited in the CWP232291 treatment group than those in the control group (**Figure 2D**). Meanwhile, the bodyweight of the two groups both remained stable (**Figure 2E**). This result further validated the tumor-suppressive function of CWP232291 in ovarian cancer.

### CWP232291 Exerts a Different Inhibitory Effect on Ovarian Cancer Organoids

**Figure 3:**
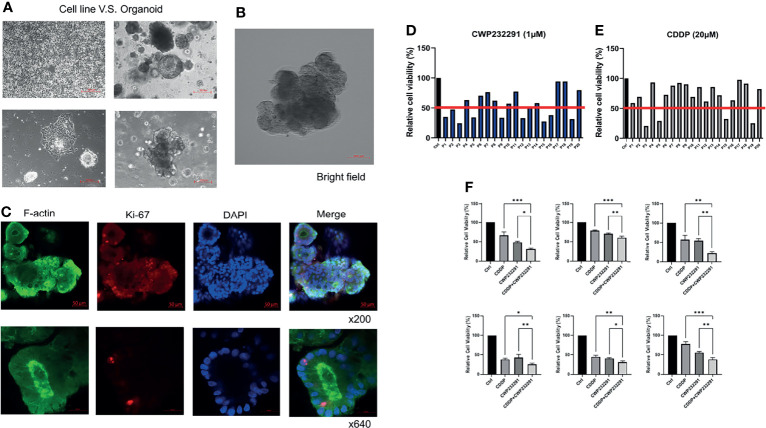
CWP232291 inhibited the growth of ovarian cancer patient-derived organoids. **(A)** Morphologic comparison of cell lines and organoids (up: low-power field; down: high-power field). **(B)** The representative morphology of organoid in bright field. **(C)** Organoid F-actin and Ki-67 staining. Twenty patient-derived organoids were established and treated with CWP232291 (1 µM) and cisplatin (20 µM). Organoids with over 50% growth inhibition were defined as sensitive. Nine of twenty (P1, P2, P3, P5, P9, P12, P15, P16, P19) was sensitive to CWP232291 **(D)** while four (P3, P5, P15, P19) of twenty was sensitive to cisplatin **(E)**. **(F)** Six organoids were treated with CWP232291 (1 µM), cisplatin (20 µM) or the combination of these two compounds. The cell viability was determined by MTT assay.CDDP: cisplatin. Results were shown as mean ± SEM (*p ≤ 0.05, **p ≤ 0.01, ***p ≤ 0.001).

Recently, 3D culture techniques including organoid culture systems have been emerging as superior models for drug sensitivity screening in cancer. In our study, we established organoids using the ovarian cancer patient-derived clinical specimens. The comparison of morphology between ovarian cancer cell lines and organoids was shown in **Figure 3A**. Besides, an organoid picture in the bright field with a more legible morphology was also presented (**Figure 3B**). In addition, the organoid was stained with the cytoskeleton marker F-actin and the proliferation marker Ki67. Pictures taken by the confocal microscopy were shown in **Figure 3C**. With the successful establishment of patient-derived organoids, we then investigated the effect of CWP232291 using 20 organoids derived from ovarian cancer tissues of different histologic subtypes. Clinical information of patients used in this study was summarized in **Supplementary Table S1**. CWP232291 (1 µM) was treated to organoids and the cell viability was determined by MTT assay after 72h. Cisplatin (20 µM) was treated as a comparison as it is one of the most commonly used drugs in ovarian cancer chemotherapy. A growth inhibition rate over 50% was defined as a good response to the drug. With this standard, 9 of 20 organoids (P1, P2, P3, P5, P9, P12, P15, P16, P19) showed a good response to CWP232291 (**Figure 3D**) while only 4 of 20 organoids (P3, P5, P15, P19) responded well to cisplatin (**Figure 3E**). Intriguingly, all 4 patients with a good response to cisplatin were also sensitive to CWP232291. Noticeably, up to 5 cisplatin-resistant patients (P1, P2, P9, P12, P16) showed a good response to CWP232291. These results provided new clues in managing patients with cisplatin resistance in clinical practice. Moreover, co-treatment of cisplatin and CWP232291 could further enhance the anti-tumor effect to varying degrees in all six organoids than individual treatment of either compound (**Figure 3F**). This implied that the combination of CWP232291 and cisplatin might be a novel therapeutic regime for ovarian cancer patients.

## Discussion

The late diagnosis and high rate of recurrence predominantly lead to the poor prognosis of ovarian cancer. Despite continuous efforts refining the scheme of debulking surgery and chemotherapy, the improvement of patient survival remains dissatisfactory. Lately, PARP inhibitors, a new class of drugs that interfere with the DNA damaging repairing abilities of tumor cells, have shed new light on the expanded treatment options for ovarian cancer patients (34, 35). Diverse clinical trials have opened a new chapter that surgical procedures and cytotoxic chemotherapy remain mainstays while targeted therapies rise promptly in ovarian cancer maintenance therapies.

The dysregulation of Wnt/β-catenin plays a fundamental role in various cancer types including ovarian cancer. Growing evidence has depicted that the altered expression of β-catenin in different subcellular locations is critical in ovarian cancer development and progression. However, it is still not clear whether the subcellular expression of β-catenin can serve as a prognostic factor in ovarian cancer. Rosen et al. have demonstrated that a low membraneous expression of β-catenin could predict the poor prognosis in endometrioid ovarian cancer (36). However, Bodnar et al. have reported an opposite result where a strong β-catenin membraneous expression was associated with the shorter OS and high chemotherapy resistance (33). Different from these two studies, Angelico et al. has illustrated that the expression of β-catenin in different subcellular localizations showed no prognostic significance in high grade serous ovarian cancer (37). On the other hand, Nagy et al. have investigated the role of β-catenin nuclear expression and found that it was positively related to favorable overall survival and platinum sensitivity (32). In our study, we showed that positive nuclear β-catenin expression was negatively correlated with the OS of ovarian cancer patients. These results suggested that the subcellular expression of β-catenin can be highly heterogeneous in different conditions and the molecular mechanism underlying β-catenin subcellular localization is greatly complicated. In addition, ovarian cancer is a heterogenous cancer type that patients with different histologic subtypes can exhibit different β-catenin expression pattern. Studies mentioned here have recruited ovarian cancer patients with disparate histologic subtypes, which might partially contribute to the inconsistent result between these studies. Besides, the relatively small sample size in most studies should also not be neglected. More large-sized and standardized studies are warranted to further investigate the role of β-catenin in ovarian cancer.

The aberrant activation of Wnt/β-catenin pathway engenders the development of multiple Wnt/β-catenin inhibitors. Therapeutic agents like Porcupine (PORCN) inhibitors, WNT ligand antagonists, and frizzled (FZD) antagonists/monoclonal antibodies have been tested in clinical trials in various cancer types (38). CWP232291 was proved to attenuate cancer growth by inhibiting β-catenin and activating ER stress pathway in castration-resistant prostate cancer (39). Currently, CWP232291 is now being tested in Phase 1 clinical trial in hematological malignancies (NCT02426723).

In this study, we demonstrated the anti-tumor effect of CWP232291 for the first time by using cell lines, patient-derived organoids and tumor xenografts. Cell line models are the most commonly used classic models in biomedical research and have been proved as a successful system for over a century. Despite that cell lines are handily maintained, less costly and relatively easy for genetic manipulation, differences between well-established cell lines and original tumors must be taken into consideration. Cell lines in two-dimensional (2D) culture are largely homogenous whereas original tumors are highly heterogeneous. Consequently, genetic and epigenetic disparities make it difficult to evaluate to what extent cell lines retain tumor original features (40). Moreover, the lack of cellular interactions in a three-dimensional (3D) environment has also constrained the translational possibility of cell line-based research. Tumor xenograft models established by engrafting human tumor tissues or cells in the host animal have also been widely used in studying cancer biology. Tumor xenografts can grow in the *in vivo* microenvironment under physiological conditions better reflecting tumor features. However, the establishment of tumor xenografts is very effortful and pricey. The ethical issues of animal experiments have always been controversial. Organoids, an *in vitro in-vivo* like system have been successfully established in various solid tumors in recent studies (41, 42). Tumor organoids better retain the genetic heterogeneity and mimic the 3D culture microenvironment, rising to be a well-applicable system for cancer research. One of the biggest advantages of organoids is the potential of identifying the optimal therapeutics for each individual patient. In our study, we first validated the tumor-suppressive function of CWP232291 using eight ovarian cancer cell lines and PA-1-derived tumor xenografts. Later, we established twenty patient tumor-derived organoids of different histologic subtypes and investigated the effect of CWP232291 and cisplatin on these organoids. According to the result, all four cisplatin-responded organoids also showed good responses to CWP232291. Meanwhile, five cisplatin-resistant organoids were sensitive to CWP232291 as well. This result was consistent with the cell line experiments where cisplatin-sensitive cell lines and cisplatin-resistant cell lines all responded well to CWP232291. Also, no signs of cross-resistance of cisplatin and CWP232291 were observed in this study. It implicates that CWP232291 might be an effective therapeutic agent in treating cisplatin-resistant or refractory ovarian cancer patients. In addition, co-treatment of cisplatin and CWP232291 could further enhance the anti-tumor effect in the organoid model, indicating there might be an additive effect of these two drugs. CTNNB1 gene mutation in ovarian cancer mainly results in the gain of function of β-catenin. This mutation is predominantly observed in the endometrioid subtype. Patients with CTNNB1 activating mutations accounted for up to 54% of all the endometrioid ovarian cancer patients (31). Our study has some limitations. For example, we mainly focus on the effect of CWP232291 using different models but there is a lack of mechanism study. And the clinical sample size used in this study is relatively small. In our further study, the underlying mechanism associated with CWP232291 response will be investigated to determine the most potentially targeted patients.

Although the mainstay of ovarian cancer treatment remains surgery and chemotherapy, recent studies have expanded the treatment options for ovarian cancer patients. Targeted therapies based on genetic changes have shifted the therapeutic landscape and upgraded the treatment algorithm. Hopefully, ovarian cancer will be transited from a fatal cancer to a chronic treatable disease in the future.

In summary, our study has first demonstrated that the small molecule CWP232291 exerts the anti-tumor effect in ovarian cancer through inhibition of β-catenin using cell lines, tumor xenografts and patient-derived organoid models. Our study has provided significant pre-clinical evidence that WNT/β-catenin inhibition would be a novel therapeutic strategy in ovarian cancer. However, more studies and clinical trials are warranted before these new therapies truly come to the forefront in clinical practice.

## Data Availability Statement

The datasets presented in this article are not readily available because it contains confidential patient data. Requests to access the datasets should be directed to wywang@snu.ac.kr.

## Ethics Statement

The studies involving human participants were reviewed and approved by Institutional Review Board of Seoul National University Hospital (IRB No. 1508-165-699). The patients/participants provided their written informed consent to participate in this study. The animal study was reviewed and approved by Seoul National University Institutional Animal Care and Use Committee (SNU-151002-1).

## Author Contributions

WW: Conceptualization, Investigation, Writing-Original Draft. UC: Conceptualization, Investigation. AY: Investigation. C-LJ: Investigation. BK: Conceptualization, Methodology. HK: Validation. JL: Validation. HJ: Validation. YH: Investigation, Writing - Review and Editing. M-HS: Methodology. J-OL: Methodology. SK: Resources. ML: Resources. J-LK: Methodology, Resources. CL: Resources. YS: Conceptualization, Writing - Review and Editing, Funding, Supervision. All authors contributed to the article and approved the submitted version.

## Funding

This research received research grants from the JW Pharmaceutical Corporation and BK21 Plus Program of the Department of Agricultural Biotechnology, Seoul National University (Seoul, Korea), a grant of Health Technology R&D Project belonging to the Korea Health Industry Development R&D Project through the Korea Health Industry Development Institute (KHIDI), funded by the Ministry of Health & Welfare, Republic of Korea (grant number: HI16C2037). WW is a recipient of the China Scholarship Council scholarship (CSC number: 201908260030).

## Conflict of Interest

Authors AY and C-LJ were employed by JW Pharmaceutical Corporation.

The remaining authors declare that the research was conducted in the absence of any commercial or financial relationships that could be construed as a potential conflict of interest.

The authors declare that this study received funding from JW Pharmaceutical Corporation. The funder had the following involvement with the study: design and performance of the mouse experiment.

## Publisher’s Note

All claims expressed in this article are solely those of the authors and do not necessarily represent those of their affiliated organizations, or those of the publisher, the editors and the reviewers. Any product that may be evaluated in this article, or claim that may be made by its manufacturer, is not guaranteed or endorsed by the publisher.

## Supplementary Material

Supplementary Table S1 Clinical information of patient samples used for IHC and organoid study.

Supplementary Table S2 Histologic subtypes of cell lines.

Supplementary Table S3 The IC50 to CWP232291 of cell lines.
